# Percept of the duration of a vibrotactile stimulus is altered by changing its amplitude

**DOI:** 10.3389/fnsys.2015.00077

**Published:** 2015-05-21

**Authors:** Eric M. Francisco, Jameson K. Holden, Richard H. Nguyen, Oleg V. Favorov, Mark Tommerdahl

**Affiliations:** ^1^Cortical Metrics, LLCSemora, NC, USA; ^2^Department of Biomedical Engineering, University of North CarolinaChapel Hill, NC, USA

**Keywords:** tactile, somatosensory, duration discrimination, amplitude discrimination, vibrotactile

## Abstract

There have been numerous studies conducted on time perception. However, very few of these have involved tactile stimuli to assess a subject’s capacity for duration discrimination. Previous optical imaging studies in non-human primates demonstrated that increasing the duration of a vibrotactile stimulus resulted in a consistently longer and more well defined evoked SI cortical response. Additionally, and perhaps more interestingly, increasing the amplitude of a vibrotactile stimulus not only evoked a larger magnitude optical intrinsic signal (OIS), but the return to baseline of the evoked response was much longer in duration for larger amplitude stimuli. This led the authors to hypothesize that the magnitude of a vibrotactile stimulus could influence the perception of its duration. In order to test this hypothesis, subjects were asked to compare two sets of vibrotactile stimuli. When vibrotactile stimuli differed only in duration, subjects typically had a difference limen (DL) of approximately 13%, and this followed Weber’s Law for standards between 500 and 1500 ms, as increasing the value of the standard yielded a proportional increase in DL. However, the percept of duration was impacted by variations in amplitude of the vibrotactile stimuli. Specifically, increasing the amplitude of the standard stimulus had the effect of increasing the DL, while increasing the amplitude of the test stimulus had the effect of decreasing the DL. A pilot study, conducted on individuals who were concussed, found that increasing the amplitude of the standard did not have an impact on the DL of this group of individuals. Since this effect did not parallel what was predicted from the optical imaging findings in somatosensory cortex of non-human primates, the authors suggest that this particular measure or observation could be sensitive to neuroinflammation and that neuron-glial interactions, impacted by concussion, could have the effect of ignoring, or not integrating, the increased amplitude.

## Introduction

Over the past several years, we have been designing sensory perceptual tests that were designed on the basis of neurophysiological observations—observed both from experiments that were conducted in our lab as well as published findings of others. For example, numerous studies have reported on the effects of repetitive vibrotactile stimulation on the SI evoked cortical response, (Cannestra et al., 1998; Chiu et al., 2005; Simons et al., 2005, 2007; Chiu, 2006) and from the findings reported in those studies, we predict that specific parametric changes in stimulus conditions could either improve or degrade sensory perceptual metrics. These perceptual findings, which proved to be robust in healthy controls (Tannan et al., 2005, 2007b; Tommerdahl et al., 2007a; Francisco et al., 2008, 2011; Zhang et al., 2011a), have demonstrated sensitivity to a number of neurological conditions (Tommerdahl et al., 2007a; Folger et al., 2008; Tannan et al., 2008; Francisco et al., 2011; Zhang et al., 2011b; Nguyen et al., 2013a,b). In other words, when a subject is neurologically compromised, the mechanisms involved in these biologically based metrics partially fail, and the neurologically compromised individual demonstrates metrics that significantly deviate from normative values.

**Figure 1:**
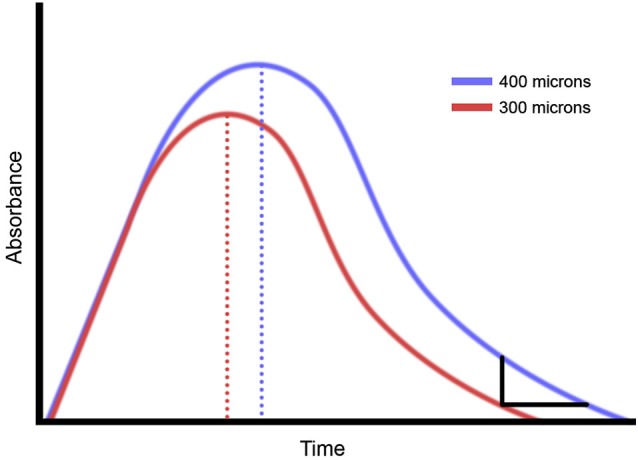
**Summary of optical intrinsic signal (OIS) imaging results**. Prior studies of non-human primate demonstrate that increasing the amplitude of a vibrotactile stimulus makes the OIS longer.

*In vivo* observations have revealed details about how sensory information is processed in the cortex, specifically that a relationship exists between time dependency of repetitive stimulation and the magnitude of stimulation. Using optical intrinsic signal (OIS) imaging, observations were made of the SI evoked response to changes in stimulus intensity (Simons et al., 2005; Chiu, 2006) and changes in stimulus duration (Chiu, 2006; Simons et al., 2007). In these studies, it was demonstrated that although absorbance values increased with increasing intensity, a center surround pattern was established and that a relationship between the contrast of the evoked SI cortical response with increases in stimulus intensity existed (Simons et al., 2007; Tommerdahl et al., 2010). The time course of the OIS response for longer duration stimuli systematically increased with stimulus duration, but perhaps more interestingly, this same time course of the OIS response also increased with increasing stimulus intensity. Figure 1 illustrates the relationship that we have observed in both the above-cited references and in unpublished experiments between stimulus intensity and the duration of the stimulus evoked OIS response for a range of durations of vibrotactile stimuli (from 500 ms to 5 s). It should be noted that with an increase in stimulus intensity, there is not only an increase in the magnitude of the evoked response, but in the duration of the response. As a result of these observations, we postulated that an increase in stimulus intensity could lead to an increase in the percept of stimulus duration. To test this idea, the duration discrimination capacity of 20 healthy subjects was obtained, and the stimulus paradigm was altered to determine if stimulus intensity would have an effect on this percept. Additionally, a pilot study was conducted to determine if the same impact of stimulus intensity on duration discrimination would be present in concussed individuals. One reason for obtaining observations from concussed individuals was to determine if duration discrimination, under different stimulus intensity conditions, was altered in this cohort. We reasoned that if neuron-glial interactions are impacted in a concussion injury, then—assuming that our above-described hypothesis was correct—it would follow that altering stimulus intensity in a duration discrimination task would have a different impact on the observations obtained from individuals from the concussed population than from healthy controls. Widespread astrocyte damage, which has long been known to occur with acute brain trauma (Chen and Swanson, 2003) would inevitably have an impact on neuron-glial interactions.

One of the fundamental questions often investigated in neuroscience is how two sensory stimuli are differentiated. The Weber function relates the difference limen (DL) in discrimination tasks to the intensity of the standard stimulus. This ratio, known as the Weber fraction, should remain constant across any standard if the sensory percept being tested obeys Weber’s Law. The Weber Fraction is usually constant for a range of stimulus intensities and can be applied to most senses, including weight, brightness, smell, frequency, contrast, velocity, and sound pitch (Cornsweet and Pinsker, 1965; Stone and Bosley, 1965; Hanna et al., 1986; Whittle, 1986; Gescheider et al., 1990, 1996, 1997; Snowden and Braddick, 1991; Stillman et al., 1993; Harris, 2005; Scholtyssek et al., 2008). Weber’s Law has been thoroughly explored in tests relying on timing perception in the auditory and visual modalities, but there remains some debate about Weber’s Law adherence in the temporal domain (particularly with a tactile stimulus).

Many studies have found significantly different Weber’s Fractions when comparing tests in the sub-second domain to tests in the macro-second domain. (0.2 and 2 s (Lavoie and Grondin, 2004), 0.2 and 1 s (Grondin, 2010), 0.5 and 3 s (Güçlü et al., 2011)). From these results, many have concluded that Weber’s Law does not apply with longer duration perceptual tasks (Getty, 1975; Bizo et al., 2006) or even in timing perception whatsoever (Blakely, 1933; Stott, 1933; Allan et al., 1971; Kristofferson and Allan, 1973; Rousseau and Kristofferson, 1973; Allan and Kristofferson, 1974; Grondin et al., 2001; Abel, 2005; Creelman, 2005). In contrast, the majority of testing finds that duration discrimination does comply with Weber’s Law within a specific range (approx. 500 ms to 2 s sources) (McGill and Goldberg, 1968; Halpern and Darwin, 1982; Rammsayer and Lima, 1991; Ehrlé and Samson, 2005; Lapid et al., 2008; Rammsayer, 2010a, 2014; Rammsayer and Ulrich, 2012). The functional relationship between DL and time has been explored extensively in the auditory and visual domains, but very few studies exist on duration discrimination in the tactile domain. This study is designed to investigate tactile duration discrimination in the sub-second to plus-second (500–1500 ms) range.

## Materials and Methods

Twenty healthy subjects (18 male, 2 female, mean age 20.5 years, SD 0.68 years) who were naïve to the study design and issue under investigation were used in this study. A survey about medication and medical history was filled out by each subject before experimental tests to exclude subjects with a history of neurological impairment. The study was performed in accordance with Declaration of Helsinki, all subjects gave their written informed consent, and the experimental procedures were reviewed and approved in advance by an institutional review board.

**Figure 2:**
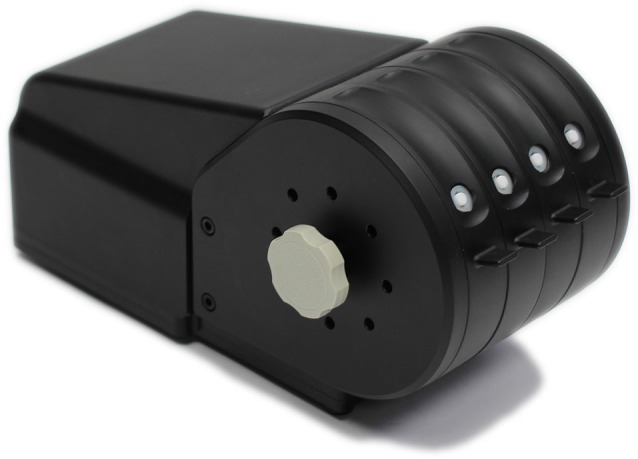
**Photo of the multi-site vibrotactile stimulator**. During an experimental session, subject was seated comfortably in a chair with right arm resting on the arm rest attached to the head unit of the stimulator. Vibrotactile stimulation was conducted via 5 mm probes that come in touch with subject’s index and middle finger.

During an experimental session, the subject was seated comfortably in a chair with his/her left arm resting on an armrest attached to the head unit of a portable four-site vibrotactile stimulator (Figure 2; CM5, Cortical Metrics, LLC; for full description of the functionally equivalent CM4, see Holden et al., 2012). Vibrotactile stimulation was conducted via 5 mm diameter probes that come in contact with subject’s digit 2 (D2; index finger) and digit 3 (D3; middle finger) of the left hand. The independent probe tips were computer-controlled and capable of delivering a wide range of sinusoidal vibrotactile stimulations of varying frequencies (measured in Hertz) and amplitudes (measured in peak-to-peak micrometers, μm). Glabrous pads of digits D2 and D3 were chosen as the test sites for two reasons: (1) to allow the convenience of access and comfort of the subject; and (2) because of the wealth of neurophysiological information that exists for the corresponding somatotopic regions of the cortex in primates. The subject’s right hand was used to indicate responses on a two-button computer mouse. During each test, the subject was instructed to indicate which finger (index/middle) perceived the longer stimulus by pressing a corresponding button on the mouse.

Visual cueing was provided with a computer monitor during the experimental runs. Specifically, an on-screen light panel indicated when the subject was to respond. An audiometer was used to make sure that no auditory cues were emitted from the stimulator during delivery of the stimuli. Practice trials were performed before each test to allow the subjects to become familiar with the test, and correct responses on 3 consecutive training trials were required before commencing with the data acquisition portion of the test. The subject was not given performance feedback or knowledge of the results during data acquisition.

### Duration Discrimination

**Figure 3:**
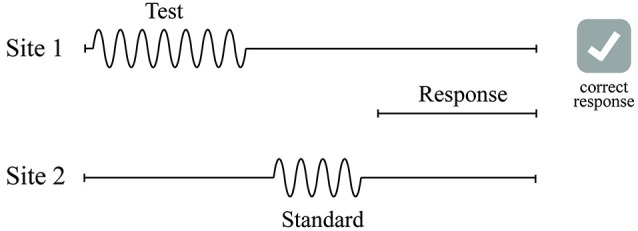
**Schematics of the duration discrimination protocol used in this study**. During each trial of the duration discrimination task, two 40 Hz vibrotactile stimuli—the standard and test—were delivered sequentially to either D2 or D3. Subject was instructed to choose the stimulus that was perceptually longer with a response box in the response interval following the two stimuli intervals.

A two-alternative forced-choice (2AFC) tracking protocol was used to evaluate the duration discriminative capacity of each subject (see Figure 3) in a manner similar to that used in a number of previous studies that have examined dual-site vibrotactile amplitude discriminative capacity (Tannan et al., 2005, 2006, 2007a,b; Tommerdahl et al., 2007a; Zhang et al., 2008). At the start of each run, the two probe tips were driven towards the skin until each tip registered a force of 0.1 g, as determined by a closed-loop algorithm in the CM-5 stimulator feedback system. The tips were then further indented into the skin by 500 μm to ensure good contact with the skin. All vibrotactile stimuli used in this study were delivered at the frequency of 40 Hz flutter.

Duration discrimination was tracked for four conditions of standard stimulus duration with each condition tracked as a separate experimental run: 500, 750, 1000, and 1500 ms. During an experimental run, a vibrotactile test stimulus was delivered sequentially either 500 ms before or after a vibrotactile standard stimulus (the standard stimulus duration remained constant throughout the run). The order (standard followed by test or test followed by standard) and loci of the stimulus was randomly selected on a trial-by-trial basis. The order of the four standards was randomized across all of the subjects. Stimulus amplitude was 300 μm. The subject was prompted on the screen of the computer to “Choose the longer duration stimulus” along with buttons labeled “Left” and “Right.” The subject selected the skin site that perceived to be the longer duration stimulus by clicking the button on the screen and a 5 s delay interval followed before onset of the next trial. The test stimulus duration began 250 ms longer than that of the standard stimulus and was increased or decreased by a 25 ms step size according to a 1-up/1-down algorithm for the first 10 trials. The subjects were unaware that one of the stimuli was of fixed duration. Correct responses resulted in the decrease of the duration of the test stimulus while incorrect responses increased the duration of the stimulus. After the initial 10 trials, the duration was varied using a 2-up/1-down algorithm. The subject’s DL was calculated by averaging the difference between the standard and the test from the final 5 trials of the 20 trial test. The rationale for implementing these algorithms was to initially expedite determination of vibrotactile discriminative range and then account for response bias; this method has been extensively reported (Tannan et al., 2006, 2007a,b; Tommerdahl et al., 2007a,b, 2008; Francisco et al., 2008; Zhang et al., 2008, 2009, 2011a,b).

### Duration Discrimination with an Amplitude Confound

The duration discriminative capacity for each subject was tested again at the 500 ms standard using 2 different amplitude confounds (see Table 1). In each of the conditions, the stimulus amplitude of the standard stimulus was either 300, 350 or 400 μm, while the amplitude of the test stimulus always remained at 300 μm. Each of the amplitudes applied to the standard (300, 350, 400 μm) were tested completely independently using three separate 20 trial 2AFC protocols pseudo-randomly interleaved in a 60 trial testing session. The 350 and 400 μm standard amplitudes were chosen to maximize the impact of the amplitude confound on the subjects discriminative capacity while maintaining the perceivable amplitude difference between the two stimuli at a minimum (350 vs. 300 μm is near a subject’s amplitude discriminative capacity (Francisco et al., 2008)). The trial in which the standard and test were 300 μm was identical to the trial in the previous section of testing with a standard duration of 500 ms. (As shown in Table 1, Condition 1 in section Duration discrimination is identical to Condition 1 in section Duration discrimination with an amplitude confound).

**Table 1:** **Testing conditions used in this study**.

		Testing Conditions		Confound Conditions	
2.1	Duration Discrimination	Test (ms)	Standard (ms)	Test Amplitude (μm)	Standard Amplitude (μm)
	Condition 1	750	500
	Condition 2	1000	750
	Condition 3	1250	1000	300	300
	Condition 4	1750	1500
**2.2**	**Duration Discrimination w. Amp. Confound**	**Test (ms)**	**Standard (ms)**	**Test Amplitude (μm)**	**Standard Amplitude (μm)**
Confound on Standard	Condition 1				300
	Condition 2	750	500	300	350
	Condition 3				400
Confound on Test	Condition 1			300
	Condition 2	750	500	350	300
	Condition 3			400
**2.3**	**Amplitude Discrimination w. Dur. Confound**	**Test (μm)**	**Standard (μm)**	**Test Duration (ms)**	**Standard Duration (ms)**
Confound on Standard	Condition 1				500
	Condition 2	400	200	500	600
	Condition 3				750
Confound on Test	Condition 1			500
	Condition 2	400	200	600	500
	Condition 3			750
**2.4**	**Concussion Pilot Study**	**Test (ms)**	**Standard (ms)**	**Test Amplitude (μm)**	**Standard Amplitude (μm)**
	Condition 1	750	500	300	300
	Condition 2				400

*Amplitudes and durations for each of the testing conditions used in this study. For each subset of testing, the order in which the conditions were presented was pseudo-randomized (Condition 1 was not necessarily the first condition presented to the subject)*.

The above described duration discrimination task was modified to determine the impact of an amplitude confound on the test stimulus. A similar 60 trial test was designed with three separate 20 trial 2AFC protocols pseudo-randomly interleaved but with three new conditions: one with a 400 μm amplitude confound located on the test stimulus, one with a 400 μm amplitude confound on the standard, and one with no confound located on either test or standard (both test and standard had an amplitude of 300 μm). All tests in this condition were simple duration discrimination tasks as described in the previous duration discrimination test; a standard of 500 ms was used and the test stimulus initiated at a duration of 750 ms while utilizing the same tracking algorithm. The subject was queried as to which stimulus was longer in the same manner as in the previously described duration discrimination task.

### Amplitude Discrimination with a Duration Confound

Amplitude discriminative capacity is defined as the minimal difference in amplitudes of two mechanical sinusoidal vibratory stimuli for which an individual can successfully identify the stimulus of larger magnitude. Discrimination capacity was assessed using a 2AFC tracking protocol that has been described and implemented in a number of previous studies (recently described by Puts et al., 2013). For all trials of amplitude discrimination, the device delivered sequential stimuli (initial stimulus parameters: 400 μm test, 200 μm standard, 40 Hz, 20 μm step size, 500 ms ISI) to D2 and D3. As seen in the previous methods, a 60 trial test was delivered with three separate 20 trial 2AFC protocols pseudo-randomly interleaved and designed to test three different test-standard duration combinations. One 20 trial set was an amplitude discrimination test in which both test and standard were delivered for equal durations of 500 ms, one set of trials had a standard stimulus duration of 600 ms (test remained at 500 ms), and one set of trials had a test stimulus duration of 600 ms (standard remained at 500 ms). The magnitude of the test stimulus was always greater than that of the standard stimulus, but the loci of the stimuli were randomly varied on a trial-by-trial basis. The subject was prompted on the screen of the computer to “Choose the stonger stimulus” along with buttons labeled “Left” and “Right.” Subjects responded by clicking the button corresponding to the digit that received the higher magnitude stimulus, and were kept naïve to the changes in durations. The same procedure was then repeated on each subject with an increase in the duration confound from 600 ms to 750 ms.

### Pilot Concussion Study

Data were collected from 19 college students who sustained a concussion (17 male, 2 female, mean age 20.1 years, SD 1.2 years), of which all were sports-related concussions (12 played football, 3 basketball, 3 soccer, and 1 lacrosse). All athletes were diagnosed with mild traumatic brain injury (mTBI) in the form of a concussion by a certified athletic trainer and the team physician with the help of the Sport Concussion Assessment Tool 2 (SCAT-2) and had no prior history of concussion or any other diagnosed medical conditions. The subjects were tested within 72 h of concussion diagnosis for both the duration discrimination and the duration discrimination with amplitude confound procedures described above (40 trial dual staircase). Two separate 20 trial 2AFC protocols were delivered in a dual staircase manner in which one set of trials were identical in amplitude (300 μm) for both test and standard stimuli, and in the other, the amplitude of the standard stimulus was 400 μm while the test stimulus was delivered at 300 μm. Individual scores post-concussion were compared to observations obtained from healthy controls.

## Results

### Duration Discrimination Follows Weber’s Law in the 500–1500 ms Range

**Figure 4:**
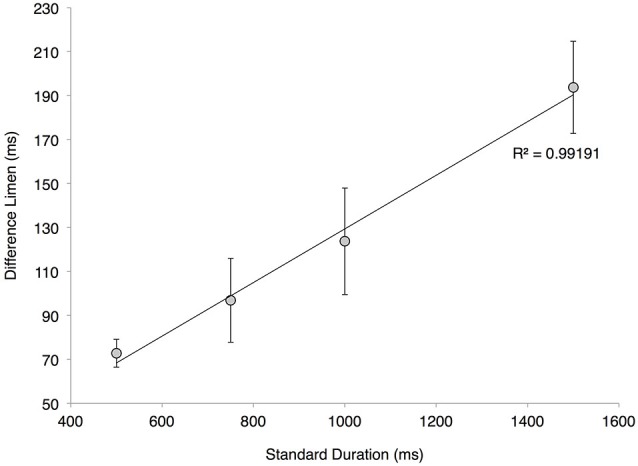
**Adherence to Weber’s Law**. Averaged difference limen (DL) values of the twenty subjects at various standard durations (with s.e. bars). The plotted linear regression has a correlation coefficient of 0.99191, a slope of 0.122 and a *y*-intercept of 7.4206.

Difference limens (DLs) obtained for duration discrimination tasks in which the standard stimulus ranged in values between 500 and 1500 ms are summarized in Figure 4. The results demonstrate that subjects performed significantly better on the duration discrimination task for shorter standard durations than for longer standard durations. In particular, the DLs increased with increasing standard durations: a DL of 72.8 ± 6.3 ms was measured with a 500 ms standard, 96.9 ± 19.1 ms for 750 ms, 123.8 ± 24.2 ms for 1000 ms, and 193.8 ± 21.0 ms for 1500 ms. A linear least-squares fit was applied to the data, and an R^2^ value of 0.992 was obtained for the linear regression (see Figure 4). The high correlation coefficient demonstrates a strong relationship between DL and the duration of the standard stimulus, thereby verifying the application of Weber’s Law for this particular discrimination task in the range of 500–1500 ms. The average measured Weber’s Fraction within the tested range was 13.1% ± 0.009.

### Increasing the Amplitude of the Standard Stimulus Relative to the Test Stimulus Increases the Difference Limen for Duration Discrimination

**Figure 5:**
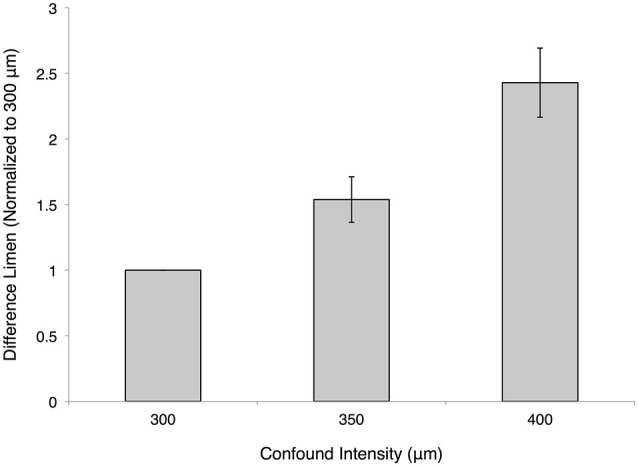
**Effect of amplitude confound on duration discrimination**. The measured difference limens (DLs) for the duration discrimination protocol are shown normalized on a subject-by-subject basis to the 300 μm condition (both test and standard are at an amplitude of 300 μm). The 350 μm confound had a normalized value of 1.537 ± 0.173 and the 400 μm confound had a normalized value of 2.428 ± 0.263 (mean ± SE).

The impact of amplitude on the percept of stimulus duration was assessed by introducing an amplitude confound into the duration discrimination task. Figure 5 illustrates that increasing the amplitude of the standard duration stimulus significantly degraded performance on the duration discrimination task. Duration discrimination DL values for each subject were normalized to unity for the 300 μm condition, while thresholds for all other conditions were normalized to within subject performance on the task. The impact of the magnitude of the stimuli on duration was thus quantified, and was statistically different from the 300 μm baseline condition: 1.54 ± 0.17 at 350 μm (*p* < 0.005, paired *t*-test) and 2.43 ± 0.26 at 400 μm (*p* < 0.0005, paired *t*-test).

### Increasing the Amplitude of the Test Stimulus Relative to the Standard Decreases the Difference Limen for Duration Discrimination

**Figure 6:**
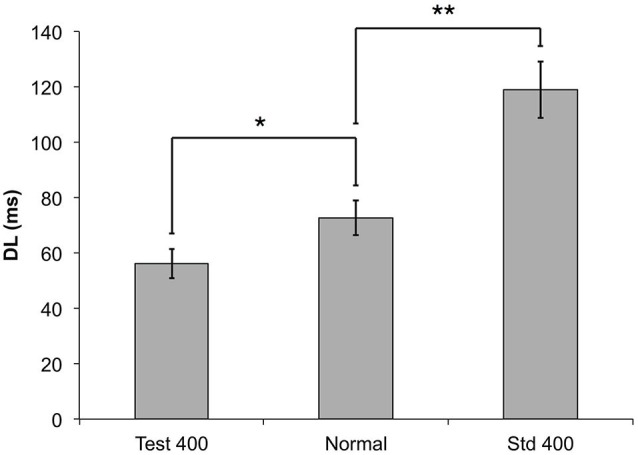
**The measured DLs for the duration discrimination protocol are compared to two different amplitude confounds**. In the Test 400 μm condition, a 400 μm stimulus was used for the test (longer) stimulus (*p < 0.05), and for the Std 400 μm condition, the 400 μm stimulus was used for the standard (shorter) stimulus (**p < 0.0005).

Figure 5 illustrates the impact of changing the stimulus amplitude of the standard when performing a duration discrimination task. Figure 6 illustrates a corollary of this finding by delivering a test stimulus with an amplitude of 400 μm while the standard stimulus has an amplitude of 300 μm. These findings suggest that an increase in amplitude on the test site improves the subjects’ ability to perform duration discrimination tasks, appreciably driving down their DL’s. The impact is not as overwhelming (or as statistically significant: *p* < 0.05, paired *t*-test) as the previously described observation with the amplitude confound on the standard. The difference between the two amplitude confounds are shown in Figure 6, and are compared to the condition with the confound completely removed (“Normal”) and the amplitudes of both test and standard delivered at 300 μm.

### There is a Reduced Impact of the Amplitude Confound on the Duration Discrimination task with Concussed Individuals

**Figure 7:**
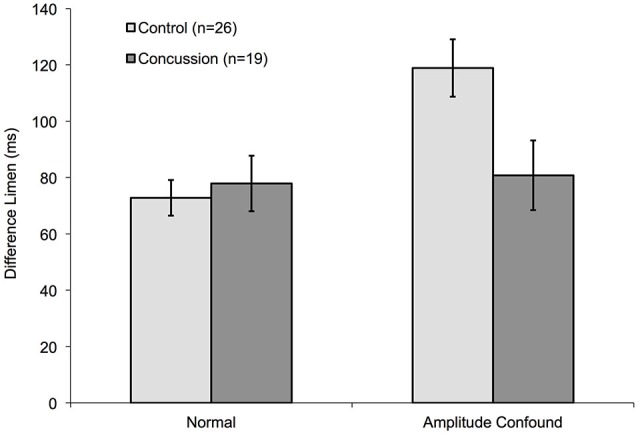
**Comparison of amplitude confound impact on concussed and non-concussed individuals**. The measured DLs for the duration discrimination protocol of control subjects from Figure 5 are compared to pilot data obtained from nineteen concussed subjects. Only the 400 μm Amplitude confound was used for this pilot study.

Although healthy controls performed significantly worse on the duration discrimination task in the presence of the amplitude confound (increased standard stimulus amplitude), concussed individuals did not appear to show a significantly different DL in the presence of the amplitude confound on the task (Figure 7). Whereas controls demonstrated an approximate 60% increase when the amplitude confound was applied, concussed individuals displayed a non-significant increase in DL of approximately 3%. A mixed-design ANOVA with one between-subjects factor (group: concussed or non-concussed) and one with-in subject factor (task: with or without amplitude confound) shows a significant main effect of task (*F* = 18.357, *p* < 0.001) and a significant group × task interaction (*F* = 21.022, *p* < 0.001). Thus, performance of the duration discrimination task in the presence of the amplitude confound appears to be better in concussed individuals than healthy controls.

### Stimulus Duration Impacts Amplitude Discriminative Capacity

**Figure 8:**
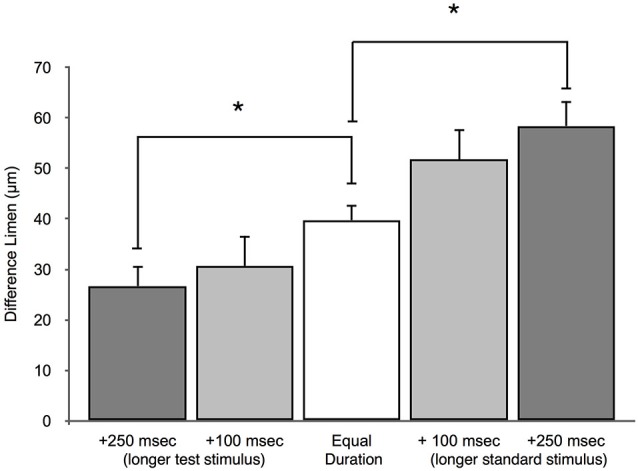
**Effect of duration confound on amplitude discrimination**. The measured DLs for the amplitude discrimination protocol are compared to two different duration confounds. In the test longer condition, the test was either +100 ms or +250 ms longer than the standard. The standard was 500 ms in these conditions. In the standard longer condition, the standard was either +100 ms or +250 ms longer than the test. The duration of the test stimulus was 500 ms for these conditions. (*p < 0.005).

An amplitude discrimination task, performed with sequential vibrations 500 ms in length, measured the average DL for control subjects to be 39.8 ± 3.4 μm for a 200 μm standard. This finding is in line with previously published amplitude discrimination values (Francisco et al., 2008). Figure 8 illustrates that when the duration of the standard stimuli was increased from 500 ms, DLs were 51.6 ± 9.2 μm when the standard was 600 ms and 58.4 ± 5.9 μm when the standard was 750 ms. Furthermore, the DLs were 30.7 ± 3.6 μm and 26.7 ± 4.9 μm when the same durations were applied to the test stimuli. These results suggest that subjects perform better on the amplitude discrimination task when the higher amplitude test stimuli are longer in duration. Amplitude discriminative capacity appears to be proportionally impacted by the length of the duration confound. The 750 ms duration confound caused statistically significant differences in DL compared to the no-confound condition when applied to both the test and the standard site (*p* < 0.005). The 600 ms condition impacted the subjects amplitude discrimination in a pattern similar to that obtained from the 750 ms trial, but with slightly less statistical certainty (*p* < 0.05).

## Discussion

This study utilized a dual-site vibrotactile duration discrimination protocol to determine the DL for a number of standard durations. The DLs were found to increase in a linear fashion with an increase in standard duration, thus adhering to Weber’s Law for the stimulus range employed in this study (500–1500 ms). Previous studies have demonstrated that amplitude discrimination capacity, obtained in a similar fashion, also follow Weber’s Law. In this study, the Weber Fraction (as a percentage) was 13.1 ± 0.9% S.E. for durations ranging from 500 ms to 1500 ms, which is consistent with what was previously reported for both tactile amplitude discrimination capacity (Francisco et al., 2008; ~13%) and for a number of reports studying auditory and visual duration discrimination (Grondin et al., 2001) ~13–14%, (Lavoie and Grondin, 2004) for 2 s (Henry, 1948; Rammsayer and Altenmüller, 2006; Rammsayer, 2014, ~14%). These results suggest that the Weber-Fechner Law holds true not only for the amplitude discrimination task but also for duration discrimination tasks with relatively high standard durations. An important finding of this study was that duration discrimination within the somatosensory system is approximately equivalent to that reported for the auditory system and for interval discrimination (Nagarajan et al., 1998). While this could suggest that discrimination of temporal information across these two sensory systems operates through a single central timing mechanism, it could also suggest that the two sensory systems task timing perception in a similar manner.

A number of studies conducted using auditory stimuli have reported inconsistent Weber-Fechner Fractions when measuring sub-second vs. macro-second epochs (Lavoie and Grondin, 2004; Grondin, 2010; Güçlü et al., 2011) or extremely long duration stimuli (>10 s, Bizo et al., 2006). The general conclusion in these papers is that this is a violation of the scalar property of time and that Weber’s Law does not hold in the temporal field. Given the results from this and a number of other recent studies (Rammsayer and Lima, 1991; Lapid et al., 2008; Rammsayer, 2010a,b; Rammsayer and Ulrich, 2012), it is more likely that the sub-second standards chosen by these studies are too small to observe the properties of Weber’s Law. The majority of studies across all sensory modalities on Weber’s Law that test a large range of standards, including those on timing, show increasing Weber-Fechner Fractions for extremely low standards. In most cases, the observed Weber Function decreases initially as it increases from a standard of zero and attains an asymptotic value for longer standard intervals. This is a common trait of almost all Weber’s Law testing. Recent studies by Lapid et al. (2008) outlined the subtle differences that different testing paradigms and presentation of the stimulus can cause in subjects’ performance, and Nagarajan et al. (1998) have shown that training/learning can have a significant impact on the subject’s ability to detect stimulus intervals. These small confounds that unsurprisingly vary from study to study could explain much of the discontinuity of Weber Fractions reported in the literature.

While the adherence to Weber’s Law for duration discrimination with tactile stimuli in this range of 500–1500 ms is a new finding, we view the observations of duration discrimination obtained with the amplitude confound as being much more significant. These observations demonstrate that when the stimulus is greater in intensity, it is perceived to be longer in duration, and it appears to be in parallel with the stimulus evoked responses observed in primary somatosensory cortex. Our previous optical imaging studies with non-human primates (Simons et al., 2005, 2007) demonstrated that larger intensity stimuli take longer to return to baseline. Additionally, the observations in the above cited optical imaging studies are also consistent with the finding in this report that increasing the duration of the stimulus has an impact on its perceived amplitude or intensity. The optical imaging signal (OIS) evoked in those studies was in the near infrared range (830 nm), and signals in that range correlate with extracellular K+ and changes in the volume of the extracellular fluid compartment attributable to glial swelling (Grinvald et al., 1991, 1994; Holthoff and Witte, 1996; Kohn et al., 1999; Vanzetta and Grinvald, 1999).

Lee et al. (2005) demonstrated that when astrocyte metabolism was inhibited with fluoracetate in sensorimotor cortical slice preparation, the optical signal was diminished, although stimulus evoked activity could still be detected neurophysiologically via evoked potential. In other words, the neural response was still viable in the absence of the glial response, and the glial response was strongly tied to the OIS that demonstrates a longer return to baseline activity with a more intense stimulus. It should also be noted that increases in amplitude of vibrotactile stimulation do not have an impact on the overall mean firing rate *post-stimulus*. This separation of neural and glial activity led the authors to hypothesize that a neuroinflammatory response could involve aberrant glial response to stimulation. If the glial response is aberrant, then the amplitude confound would be predicted to have less of an impact on a task such as duration discrimination. Obviously, this logic depends on neuron-glial interactions or integration of amplitude and duration information playing a significant role in sensory percept. If such integration plays a role in sensory percept, then it stands to reason that a neuroinflammatory process could have an impact on the process.

An alternative logic that would explain the alteration in perception of duration with increased intensity is simply cumulative firing rate. In other words, the subject would assess the overall neuronal firing that occurred across each stimulus duration that is being compared. Although the durations of the two stimuli were different in durations, overall mean firing rate is increased with stimulus intensity and the total firing across the stimulus duration with increased intensity could be perceived as longer. During the amplitude discrimination task, amplitudes are perceived as being larger with longer durations. This fits with the findings of Luna and colleagues (Luna et al., 2005) who demonstrated that increasing stimulus duration led to increases in perceived frequency, and this was correlated with increases in firing rate. In that report, the authors demonstrated that a weighted spike count covaried best with task performance.

In the case for the concussed individuals that participated in this study, timing perception (or duration discrimination) was not significantly altered. However, what was altered was the impact that an amplitude or intensity confound had on their performance of a duration discrimination task. If neuroinflammation, however elusive or ubiquitous that process, plays a role in post-concussive status, then it could be a contributing factor in the alteration of the sensory percept. Thus, an increase in neuroinflammation may result in a decrease in neuron-glial integration which could subsequently diminish the sensory illusion of perceiving a longer duration when faced with a larger amplitude stimuli that was documented in controls. Alternatively, some other aspect of sensory integration—such as integrating information between parietal and frontal cortex—could be responsible for the differences observed between concussed and non-concussed individuals. Connections from somatosensory cortex to pathways involved in timing could be disrupted with concussion, and these disruptions could conceivably lead to an inability to properly integrate information across cortical areas necessary for timing perception.

Problems in timing perception have been identified in subjects with schizophrenia (Clausen, 1950; Lhamon and Goldstone, 1956; Weinstein et al., 1958; Densen, 1977; Wahl and Sieg, 1980; Connor et al., 1990), autism (Tommerdahl et al., 2008; Kwakye et al., 2011), TBI (Schmitter-Edgecombe and Rueda, 2008; Mioni et al., 2013a,b), Parkinson’s (Sagar et al., 1988; Vriezen and Moscovitch, 1990; Artieda et al., 1992), and chronic pain (Zhang et al., 2011b; Nguyen et al., 2013a); these same conditions have also been linked to impaired glial interaction (schizophrenia (Rothermundt et al., 2004; Bernstein et al., 2009), autism (Pardo et al., 2005; Vargas et al., 2005), TBI (Di Giovanni et al., 2005; Mondello et al., 2012), Parkinson’s (Hirsch et al., 1998, 2003; Teismann et al., 2003), and chronic pain (Milligan and Watkins, 2009; Gosselin et al., 2010)). The duration discrimination metric described in this report that implements an amplitude confound measure demonstrated that subjects with concussion could be differentiated from healthy controls, and the authors believe that this metric has the potential to be used in future research to help characterize subjects with neurological conditions impacted by neuroinflammation. There are clearly additional questions to address in future longitudinal studies, such as how do multiple concusssions and/or history of concussions (either previously reported or unreported) impact task performance. The test could be more beneficial to assessing a cumulative or chronic condition, rather than acute concussion but this remains to be investigated.

Changes in stimulus evoked activity in terms of neuronal response in somatosensory cortex can neither account for, nor predict, the impact of the amplitude confound on duration discrimination capacity, and this suggests that some other mechanism is responsible for the alteration in percept that accompanies the change in stimulus amplitude. This study is an important first step in describing the integration—or lack of integration in some cases—of sensory stimuli differing in both duration and intensity. It appears that neuron-glial interactions could play a significant role in this integrative process, and if that is the case, then it stands to reason that subjects with disruptions in neuron-glial interactions, such as those with neuroinflammation, would not be impacted as severely by a stimulus condition that is normally confounded by an integrative process. Our future experimental plans include direct investigation of this interesting possibility.

## Conflict of Interest Statement

Cortical Metrics, LLC has a license from the University of North Carolina to build and distribute the vibrotactile stimulator used in this study. Mark Tommerdahl is a co-founder of the company.
